# Cannabidiol and Its Combinations with Nonsteroidal Anti-Inflammatory Drugs Induce Apoptosis and Inhibit Activation of NF-κB Signaling in Vulvar Squamous Cell Carcinoma

**DOI:** 10.3390/molecules27248779

**Published:** 2022-12-11

**Authors:** Violetta Krajka-Kuźniak, Katarzyna Papierska, Maria Narożna, Anna Jelińska, Aleksandra Majchrzak-Celińska

**Affiliations:** 1 Department of Pharmaceutical Biochemistry, Poznan University of Medical Sciences, 60-781 Poznań, Poland; 2 Program in Cell Cycle and Cancer Biology, Oklahoma Medical Research Foundation, Oklahoma City, OK 73104, USA; 3 Department of Pharmaceutical Chemistry, Poznan University of Medical Sciences, 60-780 Poznań, Poland

**Keywords:** cannabidiol, diclofenac, ibuprofen, A431, vulvar squamous cell carcinoma, inflammation, apoptosis, cell cycle, NF-κB, COX-2

## Abstract

Vulvar squamous cell carcinoma (VSCC) is a rare malignancy with a relatively good prognosis. However, the prognosis remains poor for elderly patients and those with a significant depth of tumor invasion; thus, novel treatment modalities are needed. The aim of this study was to analyze the impact of cannabidiol (CBD) and its combination with NSAIDs, diclofenac (DIC) and ibuprofen (IBU) on VSCC cells. In this regard, the MTT test was applied for cytotoxicity analysis. Moreover, the influence of CBD, DIC and IBU, as well as their combinations, on apoptosis and cell cycle distribution were analyzed by flow cytometry. The mechanisms of action of the analyzed compounds, including their impact on NF-κB signaling, p53 and COX-2 expression were evaluated using Western blot. This study shows that CBD and its combinations with NSAIDs are cytotoxic to A431 cells, but they also reduce, in a dose-dependent manner, the viability of immortalized keratinocyte HaCaT cells, and human umbilical vein cell line, EA.hy926. Moreover, the compounds and their combinations induced apoptosis, diminished the NF-κB signaling activation and reduced COX-2 expression. We conclude that CBD and its combination with DIC or IBU are promising candidates for the adjuvant treatment of high-risk VSCC patients. However, their impact on non-cancerous cells requires careful evaluation.

## 1. Introduction

Ageing is the major risk factor for cancer development, while one of the hallmarks of age-related cancerogenesis is inflammaging. Inflammaging refers to the age-related increase in chronic and systemic low-grade inflammatory processes [1]. In this regard, inflammation of the vagina and vulva, common in elderly women, increases the risk of vulvar cancer [2]. The most frequent vulvar neoplasia is vulvar squamous cell carcinoma (VSCC). It can affect a large number of organs covered by squamous epithelial lining, such as the vulva skin and the vagina [3]. The incidence of VSCC rises exponentially after 65 years of age [4]. There are two different mechanisms responsible for the development of VSCC from premalignant lesions. One of these is the human papilloma virus (HPV)-dependent pathway. It is evident that HPV, despite its known role in cervical cancer, can also trigger VSCC. HPV-independent VSCC is more common in younger women (<50 years old), usually with vulvar intraepithelial neoplasia. The HPV-independent pathway occurs more often in the elderly and results in differentiated vulvar intraepithelial neoplasia lesions and chronic inflammatory vulvar dystrophy or chronic inflammatory background, such as lichen sclerosus [3,4].

Currently, surgery maintains the main role in local and regional control of VSCC; however, radiotherapy is also frequently used. A population-based study provided by Hellman et al. reveals that the survival for stage I VSCC is excellent, but women ≥ 80 years old have a 5-fold increased risk for excess mortality [5]. Furthermore, according to this study, stage II had a 3-fold excess mortality rate compared with stage I [5]. As radical surgery can lead to reduced quality of life and morbidity, recently, a trend towards less radical locoregional surgery in vulvar cancer has been increasingly implemented [6,7]. A growing body of evidence shows that after adjustment for prognostic factors, less radical locoregional surgery has no influence on patients’ survival [6,8]. In this regard, a surgical resection margin of 2–3 mm does not appear to be associated with a higher rate of local recurrence than the widely used limit of 8 mm [8]. Nevertheless, the treatment of very old patients requires improvement [5]. Patients with very advanced age less often receive chemoradiotherapy and are treated with lower mean doses of radiation as compared to younger women [5]. The exploration of the groins is also less often performed in this group of women. Another challenging group of patients is women with synchronous lower genital tract squamous cell carcinoma. Even though rare, synchronous lower genital tract squamous cell carcinoma is associated with increased mortality compared to single-site lower genital tract squamous cell carcinoma [9]. Additionally, the prognosis of lymph node-positive patients and those with locally advanced tumors still remains poor, and novel treatment options for these women are urgently needed.

Previous studies demonstrated the important role of the nuclear factor κB (NF-κB) signaling pathway in inflammation and carcinogenesis in various tumor entities, including vulvar cancer [10,11]. In the canonical pathway, NF-κB, a heterodimeric molecule of p65 and p50 subunits, is released from inhibitory chaperones in the cytoplasm and enters the nucleus to regulate dozens of genes. In cancer cells, NF-κB integrates a range of environmental stimuli and promotes tumor progression by sustaining cell viability, inducing invasiveness and regulating metabolic adaption [12].

Besides the activation of NF-κB, the cyclooxygenase-2 (COX-2) expression is critical in cancer, as they act synergistically in promoting tumor growth, survival and resistance to chemotherapy [13]. The role of COX-2, successional prostaglandin E2 (PGE2) and its receptors have been investigated in many different tumor entities, such as colon, prostate, lung and breast cancers, as well as gynecological tumors, such as ovarian, cervical and endometrial cancers [7]. COX-2 expression plays a significant role in the evolution of vulvar lichen sclerosus to VSCC [14]. As presented by Raspollini et al., COX-2 expression in unchanged lichen sclerosus cases and lichen sclerosus cases evolving to VSCC were statistically different, and the analysis of COX-2 expression can help identify the patients with a precursor lesion that has a greater potential to evolve into VSCC [14].

Moreover, the endocannabinoid system (ECS) is involved in inflammation and pain perception, as well as the development of gynecological disorders, from fertility disorders to cancer [15]. Regarding the latter, findings from studies of gynecological cancers report that ECS is implicated in cancer cell proliferation, angiogenesis, metastasis and apoptosis [16]. Thus, it can be an attractive target for pharmacological intervention [16]. The ECS ligands are the endocannabinoids, whose actions are mimicked by exogenous cannabinoids, such as phytocannabinoids and synthetic cannabinoids [15]. Cannabinoids that act at the endocannabinoid system as specific agonists or antagonists may potentially influence dysregulation and, therefore, represent new therapeutic options for the therapy of gynecological disorders. Recently, the use of cannabidiol (CBD), the main non-psychotropic cannabinoid derived from the *Cannabis* species, has increased among cancer patients. Numerous cell culture and animal studies have demonstrated the antitumor effects of CBD in various cancer types, including glioblastoma, leukemia and breast, lung, colon and prostate cancers [17,18]. The anticancer mechanisms of CBD vary based on tumor type, ranging from cell cycle arrest to autophagy, to cell death or a combination. Additionally, CBD can also inhibit tumor migration, invasion and neo-vascularization, suggesting that CBD not only acts on tumor cells, but can also affect the tumor microenvironment, for example, by modulating infiltrating mesenchymal cells and immune cells [18]. The plethora of anti-cancer mechanisms exerted by CBD, together with a large therapeutic index, has generated interest in combining CBD with chemotherapeutic agents to improve their efficacy [19]. As far as gynecological cancers are concerned, it has been shown that CDB suppresses 3-dimensional ovarian cancer growth and may enhance the potency of classic and epigenetic therapies [20]. It has also been reported that CBD induced cervical and endometrial cancer cell death [16,21]. However, the role of CBD in VSCC remains to be elucidated.

Numerous experimental, epidemiologic and clinical studies suggest that nonsteroidal anti-inflammatory drugs (NSAIDs) are promising anti-cancer agents. However, there is a need to develop novel and safer anti-inflammatory drugs for the treatment of VSCC and other cancers. Considering the side effects of NSAIDs, the combination with CBD may become a new therapeutic solution.

Thus, in this study, we hypothesized that CBD and the combined treatment with CBD and NSAIDs might be beneficial in terms of the anti-VSCC treatment. Such an approach has not been, according to our best knowledge, evaluated so far. Therefore, the aim of this study was to analyze the impact of CBD and its combination with diclofenac (DIC) and ibuprofen (IBU) on the A431 VSCC cell line in terms of their cytotoxicity and influence on apoptosis and cell cycle distribution. The mechanisms of action of the analyzed compounds, including their impact on the NF-κB signaling pathway, p53 and COX-2 levels were also evaluated.

## 2. Results

### 2.1. CBD and NSAIDs Influence the Viability of A431, HaCaT and EA.hy926 Cells

**Figure 1:**
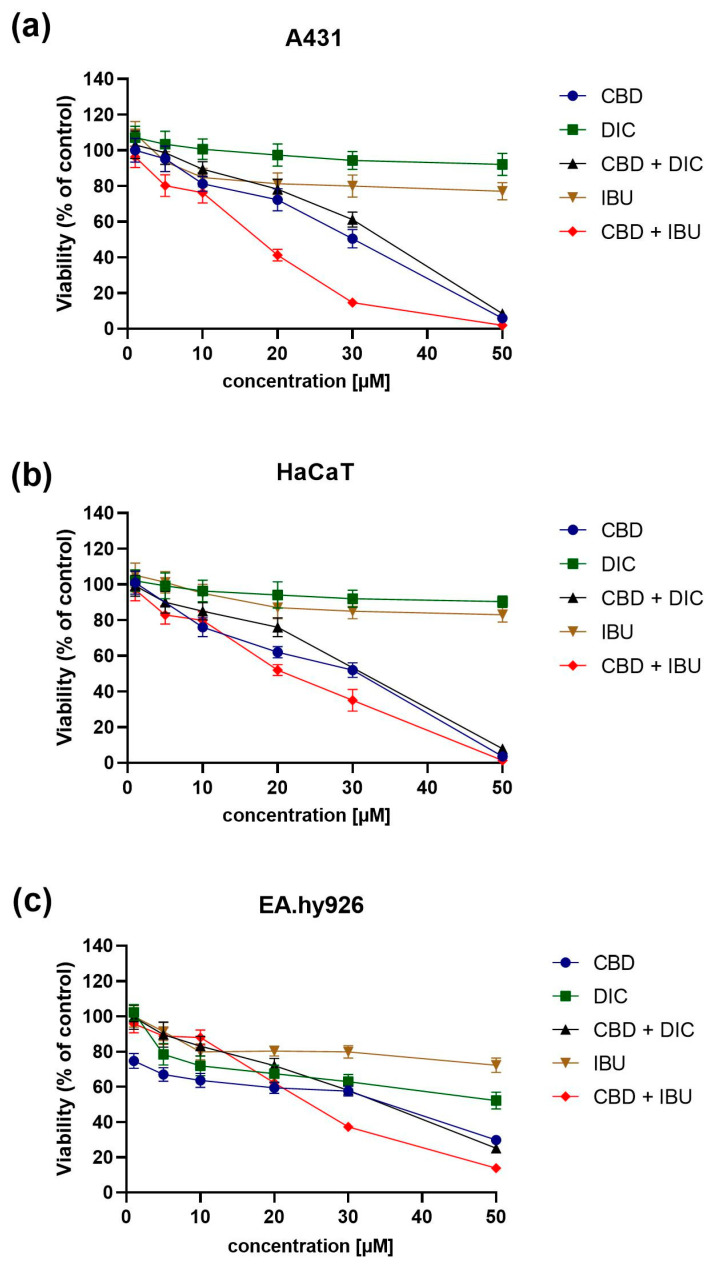
The cytotoxicity evaluation of CBD, DIC, the combination of both CBD and DIC, IBU and the combination of CBD and IBU on A431 (**a**), HaCaT (**b**) and (**c**) EA.hy926 cells. Control cells were treated with the vehicle. The values are shown as the mean ± SEM calculated from three independent experiments.

In order to analyze the impact of CBD, DIC and IBU, as well as the combination of CBD with DIC or IBU, on the viability of the A431 VSCC cell line, we performed the MTT assay after 24 h of treatment with the analyzed compounds. As reference, the immortalized human keratinocytes, HaCaT cells and human umbilical vein cells, EA.hy926 were also evaluated. The MTT assay revealed that the combination of CBD and IBU, followed by pure CBD and the combination of CBD with DIC, were the most cytotoxic, leading to complete cell death at 50 µM concentration in all three cell lines analyzed (Figure 1). Moreover, among the two NSAIDs analyzed, more cytotoxic was IBU. In contrast, the effect of DIC on the viability of A431, HaCaT and EA.hy926 cells was minimal.

The IC_50_ values, calculated based on the dose-response curves assessed by the MTT assay, are presented in Table 1.

### 2.2. CBD and NSAIDs Induce Apoptotic Cell Death in VSCC Cells, but It Is Not Accompanied by the Increased p53 Level

**Figure 2:**
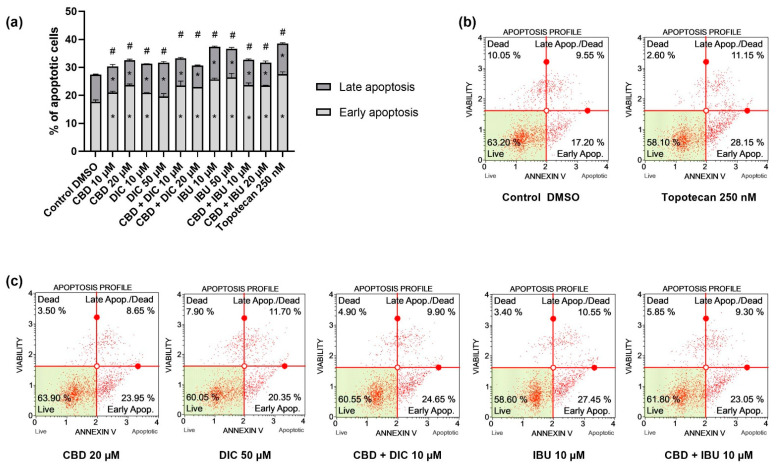
Apoptosis profile obtained using Muse ™ Annexin V & Dead Cell Assay for A431 cells treated with the analyzed compounds (CBD, DIC, IBU) and their equimolar combinations (CBD + DIC, as well as CBD + IBU). The bar chart (**a**) represents the percentage of apoptotic (early and late, as well as total apoptotic) cells after 24 h of treatment with the compounds/combination of compounds. The values are shown as the mean ± SEM calculated from two independent experiments. (*) indicates statistically significant differences from control group for early/late apoptosis, (#) above bar indicates statistically significant differences from control group for total apoptotic cells, *p* < 0.05. Apoptotic histograms of the negative and positive controls of the assay, i.e., DMSO and 250 nM topotecan, respectively, are presented in (**b**), while the representative samples are shown in (**c**). Four quadrant markers of each plot reflect the different cellular states: the upper left quadrant contains dead cells (necrosis), the upper right has late apoptotic/dead cells (cells that are positive both for Annexin V and for cell death marker 7-AAD, 7-Aminoactinomycin D), the lower left contains live cells and the lower right early apoptotic cells (cells that are positive only for Annexin V).

The flow cytometry analysis revealed that a single treatment of A431 cells with CBD, DIC or IBU, and the combi-treatment of CBD and IBU or CBD and DIC induce apoptotic cell death (Figure 2). The most significant effects were obtained after the treatment with IBU. However, the remaining compounds and combinations showed a similar effect, increasing the percentage of apoptotic cells to approximately 30%. Similar results were obtained after the treatment with our positive control, 250 nM topotecan. Overall, early, late and total apoptosis were increased after the treatment with the analyzed compounds and their combinations.

**Figure 3:**
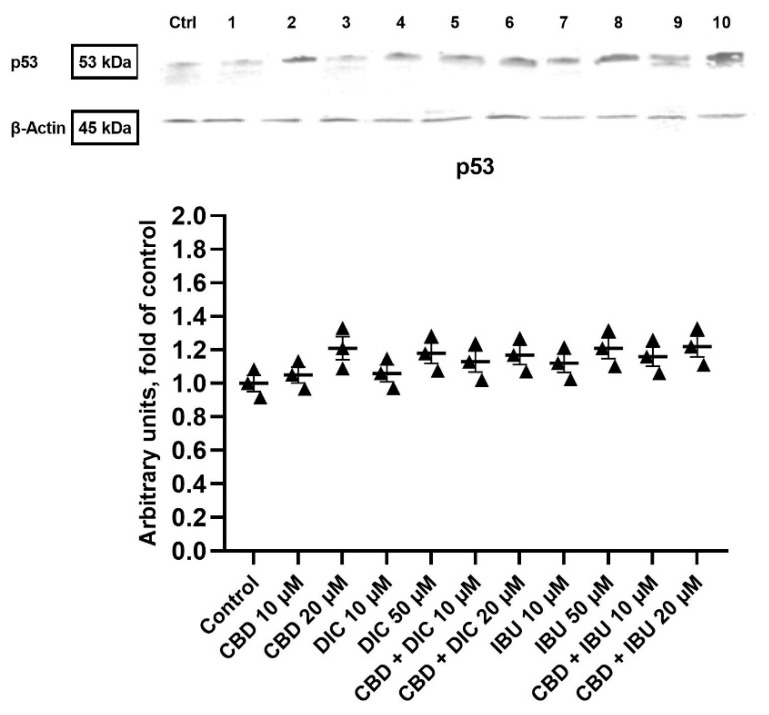
The effect of CBD and its combinations with DIC and IBU on the level of p53 protein in A431 cells. Representative Western blots are presented above the graph. The numerical code follows the order of compounds presented in charts, i.e., **Ctrl**—Control, **1**—CBD 10 µM, **2**—CBD 20 µM, **3**—DIC 10, **4** DIC—50 µM, **5**—CBD + DIC 10 µM, **6**—CBC + DIC 20 µM, **7**—IBU 10 µM, **8**—IBU 50 µM, **9**—CBD + IBU 10 µM, **10**—CBD + IBU 20 µM. The values (mean ± SEM) were calculated as a relative change in the protein level compared to control cells from three separate experiments. Original scans of blots are shown in Supplementary Materials Figure S1.

In order to understand the mechanism of action related to the pro-apoptotic effect of the studied compounds and their combinations, we analyzed the p53 protein levels. Western blot analysis revealed that after 24 h of treatment with CBD, DIC, IBU and their combinations, the p53 expression was not significantly increased and did not differ between the analyzed samples (Figure 3). Thus, the observed pro-apoptotic effects of the analyzed compounds and their combinations were not p53-dependent and suggested the involvement of an extrinsic apoptotic pathway.

### 2.3. The Distribution of Cell Cycle Phases Remains Unaltered after the Treatment with CBD and NSAIDs

**Figure 4:**
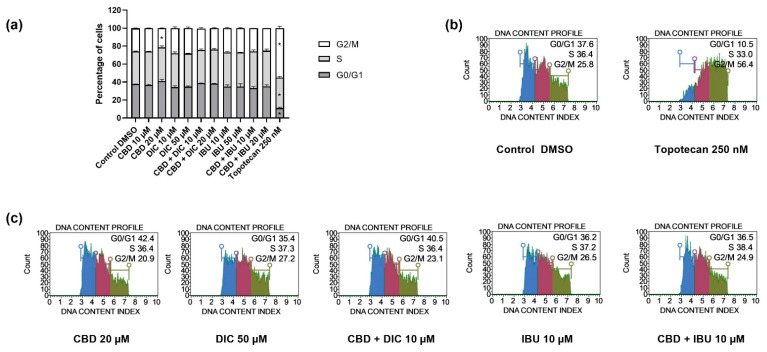
The impact of the analyzed compounds (CBD, DIC, IBU) and their equimolar combinations (CBD + DIC, as well as CBD + IBU) on the distribution of the cell cycle phases in A431 cells after 24 h treatment (**a**). The percentages of cells in the G1/G0, S and G2/M phases were analyzed by the flow cytometry after staining with propidium iodide and RNase A. The values are shown as the mean ± SEM calculated from two independent experiments. (*) indicates statistically significant differences from the control group for a particular phase, *p* < 0.05. Histograms of the negative and positive controls of the assay, i.e., DMSO and 250 nM topotecan, respectively, are presented in (**b**), while the representative samples are shown in (**c**).

To further investigate the inhibitory effect of CBD and its combination with NSAIDs on the proliferation of VSCC cells, we examined the cell cycle distribution of A431 cells after 24 h of treatment with the analyzed compounds/combinations of compounds. As presented in Figure 4, the distribution of the cell cycle phases was not altered after the treatment with the analyzed compounds; only in the case of 20 µM CBD we observed a statistically significant decrease in the number of cells in the G2/M phase. In contrast, topotecan used as a positive control of the assay, redistributed cells in all analyzed cell cycle phases, leading to G2/M cell cycle arrest.

### 2.4. The Translocation of NF-κB to the Nucleus Is Diminished after the Treatment with CBD and Its Combination with NSAIDs

**Figure 5:**
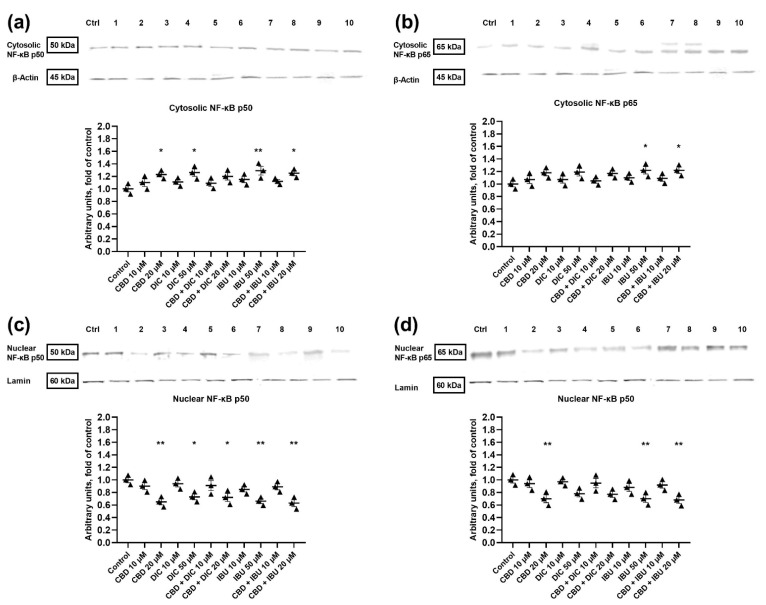
The effect of CBD and its combinations with DIC and IBU on the level of NF-κB p50 and p65 proteins (in the cytosolic fraction—panel (**a**) and (**b**); in the nuclear fraction—panel (**c**) and (**d**)) in A431 cells. Representative Western blots are presented above the graphs. The numerical code follows the order of compounds presented in charts, i.e., **Ctrl**—Control, **1**—CBD 10 µM, **2**—CBD 20 µM, **3**- DIC 10, **4** DIC—50 µM, **5**—CBD + DIC 10 µM, **6**—CBC + DIC 20 µM, **7**—IBU 10 µM, **8**—IBU 50 µM, **9**—CBD + IBU 10 µM, **10**—CBD + IBU 20 µM. The values (mean ± SEM) were calculated as a relative change in the protein level compared to control cells from three separate experiments. * *p* < 0.05 and ** *p* < 0.01 mean values significantly differed from the control group. Original scans of blots are shown in Supplementary Materials Figure S2.

In order to assess the impact of the analyzed compounds on NF-κB signaling in VSCC cells, we analyzed the cytosolic and nuclear levels of p50 and p65 NF-κB subunits (Figure 5). Western blot analysis revealed that the cytosolic level of p50 increased after the treatment with 20 µM CBD, 50 µM DIC, 50 µM IBU and an equimolar concentration of 20 µM of CBD + IBU (Figure 5a). Contrarily, the treatment with CBD, IBU, DIC and their combinations at the equimolar concentration of 20 µM significantly diminished (by 27–37%) the nuclear level of the p50 subunit (Figure 5c).

We also observed the up-regulation of the p65 subunit in the cytosol after the treatment with IBU and the combination of CBD + IBU at the equimolar concentration of 20 µM (Figure 5b). This was accompanied by a significant decrease of p65 in the nucleus after the treatment with 20 µM CBD, 50 µM IBU and a combination of CBD + IBU (equimolar of 20 µM). A trend towards down-regulation of the nuclear level of p65 was also observed after the treatment with 50 µM DIC and 20 µM CBD + DIC (Figure 5d).

### 2.5. The Activation of NF-κB Signaling Is Reduced after the Treatment with CBD and Its Combination with NSAIDs

**Figure 6:**
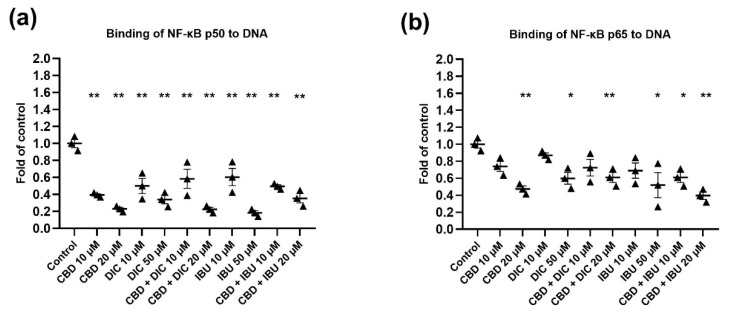
The effect of CBD and its combinations with DIC and IBU on the level of NF-κB p50 (**a**) and p65 (**b**) binding to DNA. The values (mean ± SEM) were calculated as a relative change in the protein level compared to the control cells from three separate experiments. Mean values significantly different from the control group are indicated with * *p* < 0.05 and ** *p* < 0.01.

In unstimulated conditions, inactive NF-κB dimers are sequestered in the cytoplasm by members of a family of inhibitors of κB (IκB) proteins [22]. Once an activating signal is received, the IκB proteins are rapidly phosphorylated by the ‘inhibitor of κB kinases’ (IKKs). This triggers IκB polyubiquitylation and proteasomal degradation, thereby liberating NF-κB dimers, which translocate into the nucleus, bind to their DNA targets and regulate gene expression [22]. Thus, in order to examine the activation of NF-κB, we performed the NF-κB binding assay. The results of the assessment of the binding of p50 and p65 NF-ĸB subunits to their immobilized consensus site are presented in Figure 6a,b, respectively. In this regard, binding of p50 to DNA was diminished after the treatment with all analyzed compounds and their combinations. However, the most pronounced effects were observed for 20 µM CBD (by 76%) and its combination with IBU (by 71%), as well as for 50 µM IBU (by 81%). A similar pattern, as in the case of NF-ĸB p50 DNA binding capability, was observed in the reduction of the p65 nuclear protein level (Figure 6b). In this case, 20 µM CBD and its combination with IBU resulted in the most significant reduction (by 52% and 60%, respectively) in the binding capacity of p65 to DNA.

### 2.6. COX-2 Expression Is Diminished after the Treatment with CBD and Its Combinations with DIC and IBU

**Figure 7:**
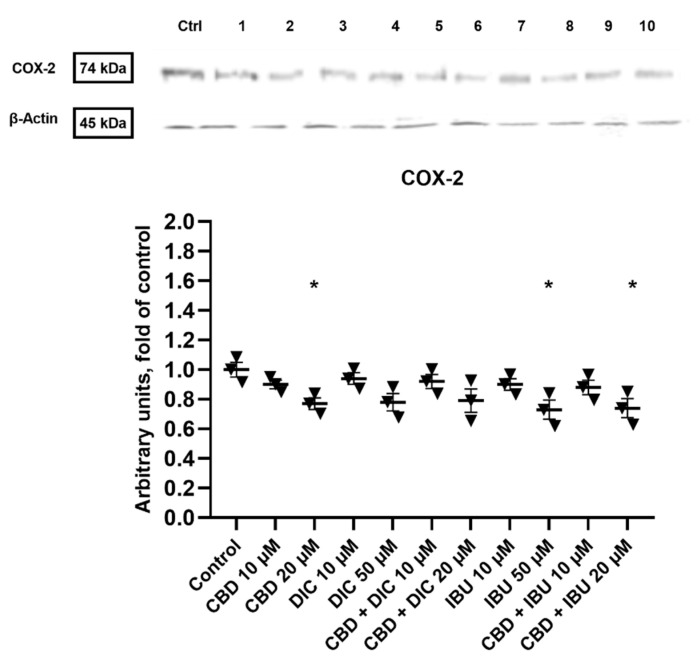
The effect of CBD and its combinations with DIC and IBU on the level of COX-2 protein in A431 cells. Representative Western blots are presented above the graph. The numerical code follows the order of compounds presented in charts, i.e., **Ctrl**—Control, **1**—CBD 10 µM, **2**—CBD 20 µM, **3**—DIC 10, **4** DIC—50 µM, **5**—CBD + DIC 10 µM, **6**—CBC + DIC 20 µM, **7**—IBU 10 µM, **8**—IBU 50 µM, **9**—CBD + IBU 10 µM, **10**—CBD + IBU 20 µM. The values (mean ± SEM) were calculated as a relative change in the protein level compared to control cells from three separate experiments. Mean values significantly different from the control group are indicated with * *p* < 0.05.

COX-2 is one of the NF-κB target genes and the critical protein in the COX-2-PGE2-EPs pathway. Thus, we wanted to analyze if the treatment with CBD and its combination with NSAIDs results in the reduction of COX-2 expression. As presented in Figure 7, the 20 µM CBD, 50 µM IBU and 20 µM CBD + IBU treatments significantly reduced the protein level of COX-2 (by 23–27%). However, a trend towards the reduction of COX-2 level was also noted for other analyzed compounds and combinations.

## 3. Discussion

VSCC is the fourth most common gynecological tumor after endometrial, ovarian and cervical cancers [23]. The therapy of elderly VSCC patients and those with a significant depth of tumor invasion remains challenging. Recently, in order to answer the needs of high-risk cancer patients, the traditional anti-cancer treatment approach, based on single-agent chemotherapy, is evolving to the form of poly-drug treatment [24]. Such ‘drug cocktails’ strengthen cancer therapies and have fewer side effects. Increasing evidence suggests that drug combinations can be more efficient in the modulation of signaling pathways involved in cancer development, including the inflammatory process [25,26,27].

NF-κB and COX-2-PGE2-EPs signaling are the central inflammatory pathways involved in gynecological carcinogenesis [28]. Thus, targeting tumor-associated inflammation is one of the valuable strategies evaluated in studies of novel anti-cancer treatments [29,30,31]. Anti-inflammatory drugs used to treat gynecological cancers are NSAIDs, e.g., celecoxib. In this regard, in preclinical studies with vulvar cancer cell lines, the COX-2 inhibitor, celecoxib, has been shown to reduce cell growth when used in combination with cisplatin [32]. Moreover, several reports indicate that NSAIDs and selective COX-2 inhibitors delay the development of endometrial cancer, ovarian cancer and cervical cancer [33]. However, COX-2 inhibitors-related cardiovascular and gastrointestinal toxicity are a serious concern; hence, finding a way to reduce their doses is of particular importance. Therefore, concerning the adverse effects of NSAIDs, it is still urgent and necessary to explore novel drug combinations and adjuvant anti-inflammatory therapies.

Natural products are considered a rich reservoir of bioactive compounds with therapeutic potentials [34]. The use of natural products as anticancer agents is an acceptable therapeutic approach due to their accessibility, applicability and their reduced cytotoxicity [25,35,36,37]. Recently, CBD, a major non-intoxicating cannabinoid in *Cannabis*, has emerged as a promising intervention for cancer research [18]. Drug combinations of CBD and chemotherapy have been explored, demonstrating that CBD enhanced the anti-tumor effects of chemotherapy at lower doses, both in vitro and in vivo [19]. In this study, we explored the idea of combining CBD with NSAIDs as a promising anticancer combination. As the impact of CBD and NSAIDs has not been evaluated deeply thus far in a model of VSCC, the present study aimed to test the influence of CBD and its combination with DIC and IBU on A431 VSCC cells. Here, we hypothesized that combining CBD with anti-inflammatory drugs might inhibit cancer cell growth to a greater extent than treatment with single drugs. The use of both compounds could also allow the reduction of doses of each individual compound, which would limit their side effects.

Our study revealed that in the concentration range of 1–50 µM, CBD and its combination with IBU and DIC reduced the viability of A431 cells in a dose-dependent manner. Moreover, the combi-treatment exerted similar (in the case of CBD + DIC) or stronger (in the case of CBD + IBU) cytotoxicity compared to the single treatment with CBD. To verify the safety of the evaluated compounds and their combinations, we also performed the MTT test on the immortalized keratinocytes, i.e., HaCaT cell line and human umbilical vein cell line, i.e., EA.hy926. Surprisingly, the effects were similar to those obtained for the cancerous cell line; thus, the safety issue for normal tissues requires further evaluation.

Gynecological cancers are characterized by the dysregulation of important cellular mechanisms, including those involved in the control of cell division, cellular differentiation and apoptosis [38]. The results of our study provided evidence that the analyzed compounds, both used as single agents and in combination, were able to induce apoptosis of A431 cells after 24 h of treatment. Nevertheless, additional apoptosis analysis with wider compound concentration ranges and more time points are needed in order to verify if synergistic or additive effects occur in the case of CBD and NSAIDs combination treatment, and if such combi-treatment is truly beneficial as compared to treatment with single compounds. Moreover, our results also indicate that the apoptosis of A431 cells induced by the treatment with CBD, DIC and IBU were not dependent on p53 activation, suggesting the involvement of the extrinsic apoptotic pathway. In A431 cells only, one allele of p53 is present; thus, the cells express only 50% of the normal p53 mRNA level [39]. We hypothesize that this might also be the reason why we did not observe significant changes in the p53 level, despite the observed pro-apoptotic effects of the analyzed compounds. Numerous reports show that CBD activates apoptosis-mediated cell death in several cancer cell lines, such as breast, colorectal, leukemia and pancreatic tumor cells; however, the reports concerning VSCC are scarce [40,41,42,43,44]. In addition, IBU and DIC were not intensively analyzed in regard to the treatment of VSCC cells. Zerbini et al. demonstrated that selected NSAIDs, i.e., sulindac sulfide and DIC, induced apoptosis and inhibited ovarian tumor growth [45]. Moreover, they provided evidence that a combination of these NSAIDs induced apoptosis and inhibited NF-κB. Indeed, several papers indicated that NF-κB signaling is a necessary factor in cancer cells survival [45,46,47]. In this study, we hypothesized that targeting NF-κB by CBD could enhance the NSAID-mediated induction of apoptosis in VSCC cells. Our flow cytometry results provide evidence that, indeed, such combinations induce apoptosis in VSCC cells, but the effect is not stronger as compared to the use of single compounds. Further studies including longer incubation times should verify if the enhanced pro-apoptotic effects can be obtained with the use of CBD and NSAIDs in regard to VSCC cells. In contrast, combinations of CBD with NSAIDs equimolar to 20 µM significantly decreased the level of NF-κB p50 and p65 proteins and their ability to bind to DNA. This suggests that these combinations effectively inhibit NF-κB in VSCC cells.

Inhibition of COX-2 provides a high possibility to exert therapeutic outcomes in cancer cells. Moreover, administration of COX-2 inhibitors in a preoperative setting could reduce the risk of metastasis in cancer patients. COX-2 inhibition also sensitizes cancer cells to treatments, such as radio- and chemotherapy [48,49]. Taking into consideration that inflammation is a key event during VSCC tumorigenesis [50], we analyzed the protein level of COX-2 after the treatment with the analyzed compounds. Importantly, we noticed a significant decrease in COX-2 levels after the treatment with 20 µM CBD. Additionally, we detected a significant decrease in the level of COX-2 after the treatment with 50 µM IBU and the combination of CBD and IBU, as compared to the control group. Similar to our results, the inhibition of cancer progression through the modulation of the distorted tumor-associated inflammation with NSAIDs was also reported in endometrial, ovarian and cervical cancer lines [51,52,53].

To conclude, our study demonstrated that CBD and its combination with DIC and IBU exert anti-cancer activity in A431 cells by inhibiting NF-κB and COX-2 signaling pathways. As high-risk VSCC patients could benefit from adjuvant locoregional anti-inflammatory therapy, we suggest that CBD and DIC, as well as CBD and IBU, should be further evaluated in this context. Additionally, patients with a high risk of tumor recurrence or with significant depth of invasion and lymph node-positive ones could take advantage of such an adjuvant treatment. However, the safety of such drug combinations in regard to non-cancerous tissues requires further investigation.

## 4. Materials and Methods

### 4.1. Cell Culture and Viability Assay

Human VSCC A431 cells and immortalized human keratinocyte HaCaT cells were obtained from the European Collection of Authenticated Cell Cultures (ECACC) and Cell Lines Service (CLS, Eppelheim, Germany), respectively. Moreover, human umbilical vein cell line, EA.hy926 was obtained from American Type Culture Collection (ATCC, Manassas, VA, USA). All 3 cell lines were maintained in Dulbecco’s Modified Eagle’s Medium (DMEM, Sigma-Aldrich, St. Louis, MO, USA ) containing 10% fetal bovine serum (FBS, EURx, Gdańsk, Poland) and 1% antibiotics solution (Sigma-Aldrich, St. Louis, MO, USA) at 37 °C, in a humidified 5% CO_2_ atmosphere.

Cannabidiol (CBD) was purchased from Medcolcanna Organics Inc. (Calgary, Canada), and diclofenac sodium (DIC) and ibuprofen (IBU) were purchased from POL-AURA (Olsztyn, Poland).

The effect of the analyzed compounds and combinations on cell viability was assessed using MTT assay, following the standard protocol. Briefly, A431, HaCaT and EA.hy926 cells were seeded (10^4^ per well) in 96-well plates. After 24 h of pre-incubation in a complete medium, compounds were added in various concentrations, and cells were incubated for 24 h. The DMSO concentration did not exceed 0.1%. Later, cells were washed twice with phosphate-buffered saline (PBS) and further incubated for 4 h with a medium containing 0.5 mg/mL 3-(4,5-dimethylthiazol-2-yl)-2,5-diphenyl-2H-tetrazolium bromide (MTT). Then, the formazan crystals were dissolved in acidic isopropanol, and the absorbance was measured at 570 nm and 690 nm. All experiments were repeated three times.

To assess the effect of CBD and its combination with selected NSAIDs on measured parameters, 1 × 10^6^ cells were seeded per 100 mm culture dish, and, after 24 h of initial incubation, the cells were treated with 10 µM, 20 µM CBD, and 10 µM, 50 µM DIC and, 10 µM, 50 µM IBU, and its combinations: with IBU (10 µM, 20 µM) and, its combination with DIC (10 µM, 20 µM) and 0.1% vehicle (DMSO, dimethyl sulfoxide) control. Incubation lasted 24 h, and the cells were harvested.

### 4.2. Apoptosis Analysis

The Muse^®^ Annexin V & Dead Cell Kit (Merck, Darmstadt, Germany) was used in the evaluation of the apoptosis, according to the manufacturer’s protocol, using one of the known apoptosis markers—the externalization of phosphatidylserine. Additionally, the 7-Aminoactinomycin D (7-AAD) staining enables discrimination between early and late apoptotic cells. Briefly, cells (3 × 10^5^ per well) were seeded in 6-well plates, pre-incubated for 24 h and further grown for 24 h in the presence of the tested compounds and topotecan (0.5 µM), which was used as a positive control of the induction of apoptosis. After incubation, cells were harvested by trypsinization, resuspended in fresh medium, stained with Annexin V and 7-AAD solution, subjected to 20 min incubation in the dark at room temperature and analyzed by flow cytometry on a Muse^®^ Cell Analyzer. Data were evaluated using Muse^®^ 1.5 Analysis Software.

### 4.3. Cell Cycle Assay

The Muse^®^ Cell Cycle Kit (Merck, Darmstadt, Germany) was used in the determination of cell cycle, according to the manufacturer’s protocol. The cell cycle distribution analysis was performed by propidium iodide staining, and its specificity in binding to DNA was improved by the addition of RNase A. Briefly, cells (3 × 10^5^ per well) were seeded in 6-well plates, pre-incubated for 24 h, and further grown for 24 h in the presence of the tested compounds. Topotecan (0.1 µM)-treated cells were used as a positive control of the cell cycle arrest. Subsequently, cells were harvested, fixed in ice-cold 70% ethanol and frozen until further analysis at −20 °C. The next day, fixed cells were collected, washed with PBS buffer and stained with the mixture of reagents 30 min before flow cytometry analysis on a Muse^®^ Cell Analyzer. Data were evaluated using Muse^®^ 1.5 Analysis Software.

### 4.4. Nuclear, Cytosolic and Total Protein Lysates Preparation

The subcellular extracts were prepared using the Nuclear/Cytosol Fractionation Kit (BioVision, Milpitas, CA, USA). Lysates were prepared using Radioimmunoprecipitation assay (RIPA) buffer with the addition of protease inhibitors (Sigma-Aldrich, St. Louis, MO, USA). The Lowry method assessed the concentration of proteins, and then samples were stored at −80 °C until further analysis.

### 4.5. Western Blot Analysis

Cell lysates (for COX-2 and p53), cytosolic and nuclear extracts (for NF-κB p50, NF-κB p65) were separated on 12% and/or 10% SDS-PAGE slab gels and proteins were transferred to nitrocellulose membranes. After blocking for 2 h with 10% skimmed milk, proteins were probed with rabbit polyclonal anti-NF-κB p50, rabbit polyclonal anti-NF-κB p65, rabbit polyclonal anti-COX-2, mouse monoclonal anti-p53, rabbit polyclonal anti-β-actin and rabbit polyclonal anti-lamin antibodies (Santa Cruz Biotechnology, Dallas, TX, USA). β-actin and lamin expression served as loading controls. Horseradish peroxidase (HRP)-conjugated anti-rabbit IgG or anti-mouse IgG (Boster Bio, Pleasanton, CA, USA) secondary antibodies were used in the staining reaction. Bands were visualized by the chemiluminescent HRP substrate of the Clarity ECL Kit (Bio-Rad Laboratories, Hercules, CA, USA ). The amount of immunoreactive product in each lane was determined using Quantity One software (BioRad Laboratories, USA). Values were calculated as relative absorbance units (RQ) per mg protein. All the experiments were repeated three times.

### 4.6. NF-ĸB Binding Assay

NF-κB p50, NF-κB p65 activation was assessed by enzymatic immunoassay (Transcription Factor ELISA Assay Kit, Active Motif, La Hulpe, Belgium), according to the manufacturer’s instructions. Activated NF-ĸB was measured as the amount of p65 and p50 subunits contained in the DNA-binding complex. The oligonucleotides containing (5′-GGGACTTTCC-3′) a consensus site for NF-κB were immobilized in/on microplates as bait. Nuclear fractions were incubated with oligonucleotides for one hour, then wells were washed and DNA-bound subunits were detected by the specific primary antibody and secondary antibody conjugated with HRP. The results were expressed as the normalized level of absorbance (OD450 nm per mg of protein).

### 4.7. Statistical Analysis

Statistical analysis was performed using GraphPad Instat version 3.10 (GraphPad Software, San Diego, CA, USA). The data are presented as the means ± SEM. To assess the significance of the changes in the evaluated parameters, one–way ANOVA with Dunnett’s post hoc test and Student *t*-test were performed with significance levels of *p* < 0.05 and *p* < 0.01.

## 5. Conclusions

The results of our study regarding the use of a CDB and NSAIDs, as well as the combi-treatment of CBD together with NSAIDs, provide the foundation for a new approach to therapy of VSCC. Further in-depth investigation of these drug combinations is needed to maximize antitumor efficacy and minimize the side effects, especially in regard to non-cancerous cells. Eventually, we suggest that together with novel treatment modalities of VSCC treatment, including the use of immune-response modulators, anti-viral agents, photodynamic therapy and prophylactic HPV vaccination, CBD and NSAIDs should be further analyzed as adjuvant locoregional anti-inflammatory therapy of VSCC [54].

## Figures and Tables

**Table 1:** The IC_50_ values for a 24 h treatment of A431, HaCaT and EA.hy926 cell lines with CBD, DIC, the combination of CBD + DIC, IBU and the combination of CBD + IBU.

Compound/Combination	A431 [IC_50_ ± SEM (µM)]	HaCaT [IC_50_ ± SEM (µM)]	EA.hy926 [IC_50_ ± SEM (µM)]
CBD	30.0 ± 0.28	31.0 ± 1.94	35.5 ± 3.24
DIC	>50	>50	>50
CBD + DIC	34.0 ± 1.4	37.5 ± 3.86	35.0 ± 1.65
IBU	>50	>50	>50
CBD + IBU	17.5 ± 2.69	31.0 ± 2.98	25.0 ± 0.61

## Data Availability

The data supporting reported results can be found in the Poznan University of Medical Sciences databases.

## Supplementary Materials

The following supporting information can be downloaded at: https://www.mdpi.com/article/10.3390/molecules27248779/s1, Figure S1. The representative immunoblots respectively for Figure 4. Panel a and b—The level of NF-κB p50 and p65 protein in the cytosolic fraction. Panel c and d—The level of NF-κB p50 and p65 protein in the nuclear fraction. Data were normalized against the level of β-actin (cytosolic proteins) or lamin (nuclear proteins). Ctrl—Control, 1—CBD 10 µM, 2—CBD 20 µM, 3—DIC 10, 4 DIC—50 µM, 5—CBD + DIC 10 µM, 6—CBC + DIC 20 µM, 7—IBU 10 µM, 8—IBU 50 µM, 9—CBD + IBU 10 µM, 10—CBD + IBU 20 µM. Figure S2. The representative immunoblots respectively for Figure 6. The level of COX-2 protein. Data were normalized against the level of β-actin. Ctrl—Control, 1—CBD 10 µM, 2—CBD 20 µM, 3—DIC 10, 4 DIC—50 µM, 5—CBD + DIC 10 µM, 6—CBC + DIC 20 µM, 7—IBU 10 µM, 8—IBU 50 µM, 9—CBD + IBU 10 µM, 10—CBD + IBU 20 µM. Figure S3. The representative immunoblots respectively for Figure 7. The level of p53 protein. Data were normalized against the level of β-actin. Ctrl—Control, 1—CBD 10 µM, 2—CBD 20 µM, 3—DIC 10, 4 DIC—50 µM, 5—CBD + DIC 10 µM, 6—CBC + DIC 20 µM, 7—IBU 10 µM, 8—IBU 50 µM, 9—CBD + IBU 10 µM, 10—CBD + IBU 20 µM.
