# Supplementary material for: Cannabidiol and Its Combinations with Nonsteroidal Anti-Inflammatory Drugs Induce Apoptosis and Inhibit Activation of NF-κB Signaling in Vulvar Squamous Cell Carcinoma
Source: Molecules. 2022 Dec 11;27(24):8779. doi: 10.3390/molecules27248779 (PMC9781989; doi:10.3390/molecules27248779)
Supplement: Supplementary file 1 [file molecules-27-08779-s001.zip › molecules-2047105-supplementary.pdf]

Supplementary Figures

Article

CANNABIDIOL AND ITS COMBINATIONS WITH NONSTEROIDAL ANTI-INFLAMMATORY DRUGS INDUCE APOPTOSIS AND INHIBIT ACTIVATION OF NF-KB SIGNALING IN VULVAR SQUAMOUS CELL CARCINOMA

Violetta Krajka-Kuźniak[1]*, KatarzynaPapierska[1], Maria Narożna[2], Anna Jelińska[3] and Aleksandra Majchrzak-Celińska[1]

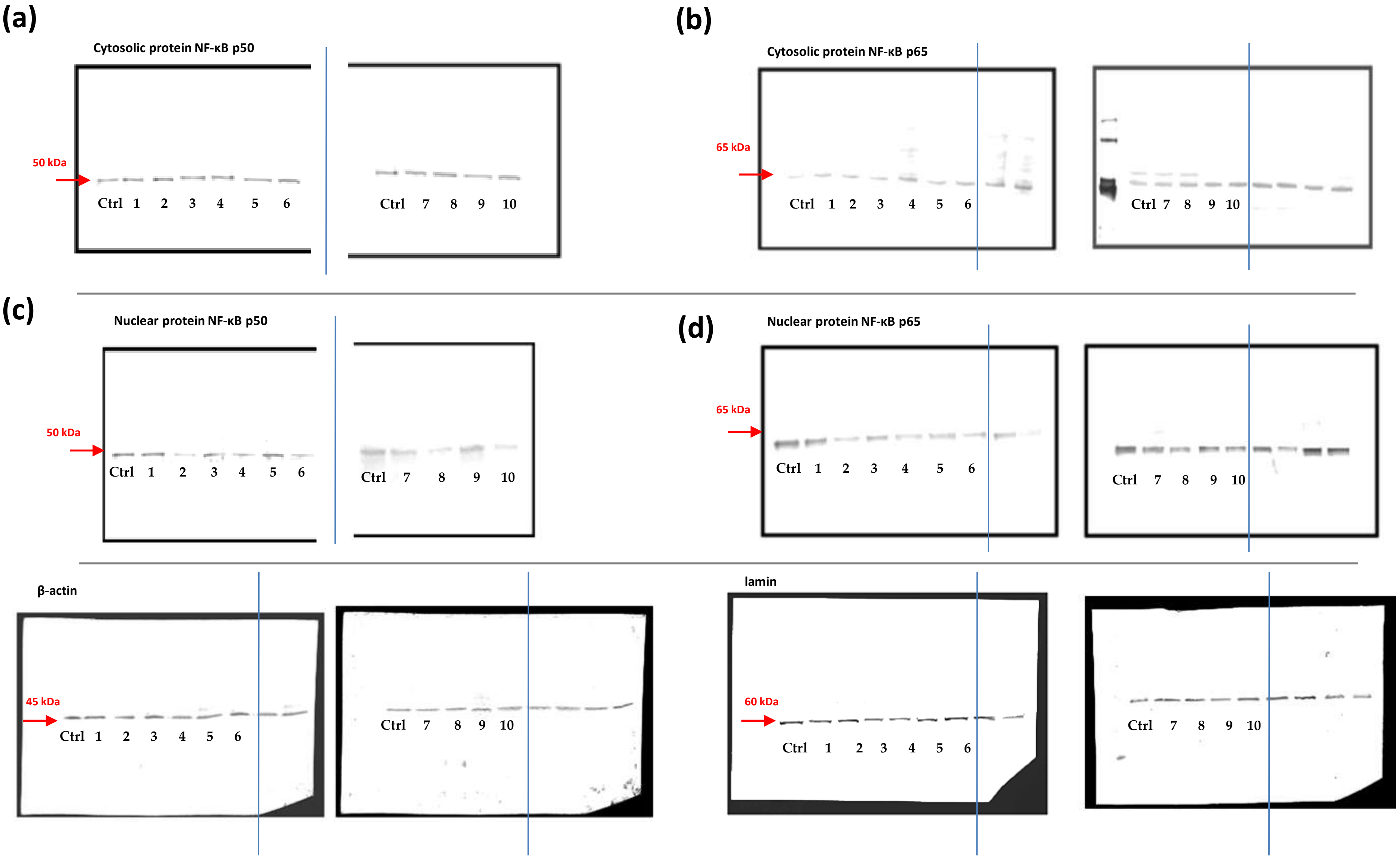

Figure S1. The representative immunoblots respectively for Figure 4. ***Panel a** and **b** - The level of NF-κB p50 and p65 protein in the cytosolic fraction.* ***Panel c** and **d** - The level of NF-κB p50 and p65 protein in the nuclear fraction.* Data were normalized against the level of β-actin (cytosolic proteins) or lamin (nuclear proteins). **Ctrl –** Control, **1** - CBD 10 μM, **2** - CBD 20 μM, **3**- DIC 10, **4** DIC – 50 μM, **5** – CBD + DIC 10 μM, **6** – CBC + DIC 20 μM, **7** – IBU 10 μM, **8** – IBU 50 μM, **9** – CBD + IBU 10 μM, **10** – CBD + IBU 20 μM.

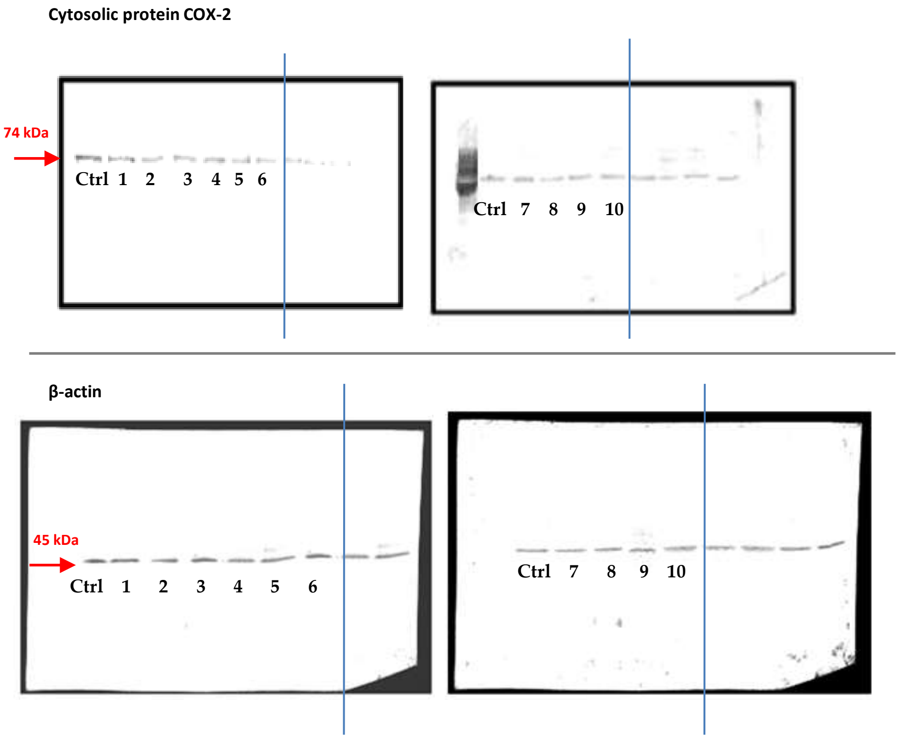

Figure S2. The representative immunoblots respectively for Figure 6. *The level of COX-2 protein*. Data were normalized against the level of β-actin. **Ctrl –** Control, **1** - CBD 10 µM, **2** - CBD 20 µM, **3**- DIC 10, **4** DIC – 50 µM, **5** – CBD + DIC 10 µM, **6** – CBC + DIC 20 µM, **7** – IBU 10 µM, **8** – IBU 50 µM, **9** – CBD + IBU 10 µM, **10** – CBD + IBU 20 µM.

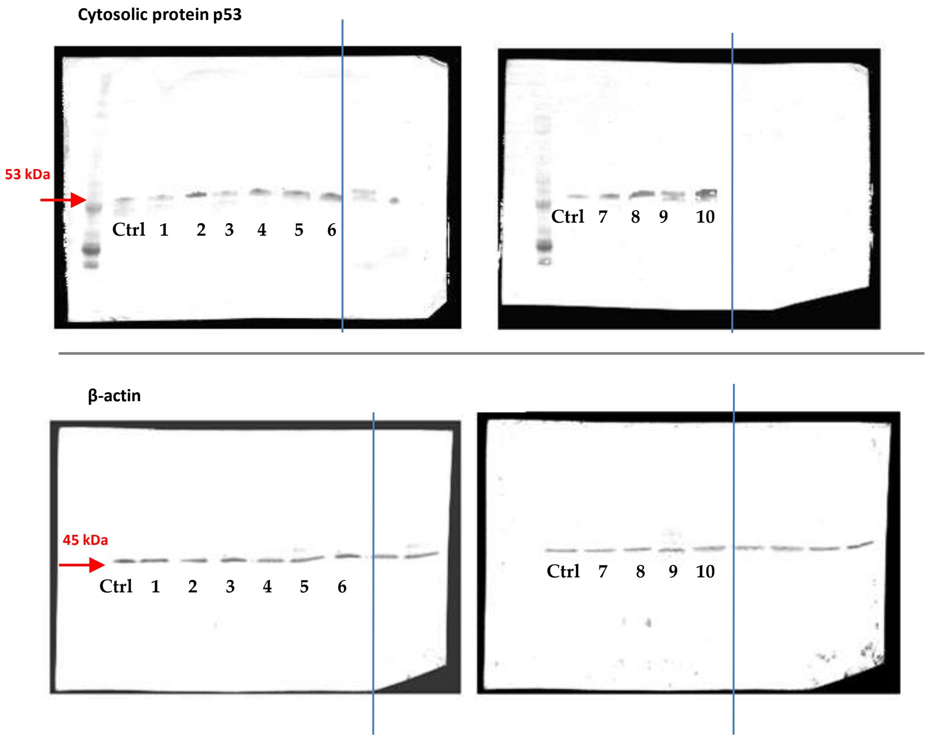

Figure S3. The representative immunoblots respectively for Figure 7. *The level of p53 protein.* Data were normalized against the level of β-actin. **Ctrl –** Control, **1** - CBD 10 µM, **2** - CBD 20 µM, **3**- DIC 10, **4** DIC – 50 µM, **5** – CBD + DIC 10 µM, **6** – CBC + DIC 20 µM, **7** – IBU 10 µM, **8** – IBU 50 µM, **9** – CBD + IBU 10 µM, **10** – CBD + IBU 20 µM.
